# College students’ psychology and behavior in the context of online public opinion: a cross-sectional study in Jiangsu Province, China

**DOI:** 10.3389/fpsyg.2024.1475581

**Published:** 2024-11-26

**Authors:** Xiaoqing Chen, Qi Wu, Dehui Yin

**Affiliations:** ^1^Department of Student Affairs, Xuzhou Medical University, Xuzhou, China; ^2^Center for Medical Statistics and Data Analysis, School of Public Health, Xuzhou Medical University, Xuzhou, Jiangsu, China; ^3^Xuzhou Engineering Research Innovation Center of Biological Data Mining and Healthcare Transformation, School of Public Health, Xuzhou Medical University, Xuzhou, China; ^4^Jiangsu Engineering Research Center of Biological Data Mining and Healthcare Transformation, School of Public Health, Xuzhou Medical University, Xuzhou, China

**Keywords:** public opinion, college students, psychology, network media, behavioral performance

## Abstract

**Background:**

With the rapid development of the Internet and the widespread use of social media, online public opinion has profoundly impacted the psychology and behavior of college students. College students are in a crucial stage of psychological development and self-awareness, making them highly sensitive to online information and easily influenced by online public opinion.

**Methods:**

This study employed a cross-sectional design to explore the psychological adaptation and behavioral responses of college students to online public opinion. Data were collected from a convenience sample of 2,294 college students across four universities in Xuzhou City, Jiangsu Province, using an online questionnaire administered via Questionnaire Star. The study utilized three well-established scales: the Belief in a Just World (BJW) Scale, the Connor-Davidson Resilience Scale (CD-RISC), and the Internet Altruistic Behavior (IAB) Scale. Statistical analyses were conducted using SPSS 26.0, encompassing descriptive statistics, independent samples t-tests, ANOVA, correlation analysis, and multiple linear regression analysis, with a significance level set at *p* < 0.05. Any responses with missing or inconsistent data were excluded from the analysis, ensuring a final effective response rate of 95.7%.

**Results:**

Among the 2,294 participants, 60.1% were female, 56.8% were from rural areas, and 57.4% were non-only children. Univariate analysis showed significant relationships between BJW scores and gender, family economic status, parents’ attitudes, relationships with classmates, and emotional responses to negative online content (*p* < 0.001). CD-RISC scores were significantly related to only child status, family economic status, parents’ attitudes, relationships with classmates, and emotional responses to negative online content (*p* < 0.05). IAB scores were significantly related to gender, grade level, family economic status, parents’ attitudes, relationships with classmates, and emotional responses to negative online content (*p* < 0.05). Correlation analysis revealed significant associations among BJW, CD-RISC, and IAB. Multiple regression analysis identified key predictors for each scale, including gender, parents’ attitudes, relationships with classmates, emotional responses to negative online content, and various other factors (*p* < 0.001).

**Conclusion:**

In the context of online public opinion, targeted interventions by families and schools are needed to regulate the psychological and behavioral states of college students, promoting good mental health and positive behavior in the complex online environment.

## 1 Introduction

In the age of the Internet and rapid 5G technological advancements, the use of computers and smartphones has become an integral part of daily life. Traditional media, such as newspapers and radios have gradually lost their dominant position in shaping public opinion. Online platforms now allow individuals to share their thoughts freely, leading to a more democratized flow of information (Zhang et al., 2022). University students, in particular, operate with greater freedom and autonomy in this digital landscape (Jiang et al., 2023). They can access primary information sources online with ease and actively express their perspectives, making them key contributors to the creation and evolution of online public opinion. However, because they are in a transitional phase of integrating into society, with values still in formation, they can be easily influenced or misled in environments rich with online discourse and freedom of expression. Thus, understanding the psychological and behavioral patterns of university students in relation to online public opinion is of critical importance. Psychologists have thus extended their research to digital domains to investigate how online interactions influence psychological states and behaviors (Hongxia et al., 2021; Xianliang et al., 2021).

The belief in a just world (BJW) is regarded as a crucial psychological resource that enhances individual survival and developmental prospects by serving dual adaptive and motivational functions (Dalbert, 2001). Adaptively, BJW helps maintain healthy behaviors and cognitive patterns. Dalbert and Stoeber (2006) found that BJW can predict an individual’s subjective well-being. A belief in a just world instills a sense of security and control, affecting cognition, actions, and emotional responses. Some studies suggest that individuals with strong BJW are better equipped to handle societal events and crises, demonstrating enhanced coping mechanisms and adaptability (Dalbert, 2001). Motivationally, BJW fosters the expectation of fair treatment, encouraging just actions toward others, compliance with social norms, and the cultivation of harmonious interpersonal relationships (Lerner, 1980). In the context of online public opinion, university students’ BJW can serve as a psychological support resource, influencing their interpretation and reaction to online information. This belief helps students remain calm and rational in a volatile online environment, better coping with the challenges and confusion posed by online public opinion.

Resilience is a personality trait characterized by the ability to overcome adversity and return to normal functioning (Connor and Davidson, 2003), as well as adapt well to life’s adversities, traumas, tragedies, threats, or significant stress. It refers to the dynamic developmental process individuals undergo when facing life events and setbacks (Scheffers et al., 2022). Existing research indicates that cyber victimization is a risk factor impacting resilience levels (Oydemir and Dikmen, 2024). Individuals coping with the perception of risk arising from online public opinion are highly susceptible to bring negative emotions to themselves (Skagerlund et al., 2020). Studies have found that social media use is closely related to the mental health of Chinese university students, offering connectivity and support, but also potentially facilitating social comparison and the stigmatization of mental illnesses (Zhang, 2024). In the context of online public opinion, students are more susceptible to external influences, particularly negative information, which can undermine their resilience.

Altruistic behavior refers to actions taken without self-serving motives that provide help and benefits to others (Shaw, 1991), also known as prosocial behavior. As a positive behavioral quality, altruism can enhance one’s sense of meaning and happiness in life (Moynihan et al., 2015; Van Tongeren et al., 2016). Essentially, online altruism is no different from offline altruism; it involves consciously helping others in the internet environment, representing a form of positive online behavior (Xianliang et al., 2021; Wright and Li, 2011).

A review of past literature reveals that researchers tend to select university students as subjects when studying positive online behaviors (such as altruism) (Zheng et al., 2022; Zhang et al., 2021; Wang et al., 2023). This is likely because, on current online platforms, university students possess the physical vigor of youth, the emerging adulthood that stimulates their sense of social responsibility, and a high frequency of activity that positions them as the main users of the Internet in researchers’ eyes. For example, in academic contexts, students share study resources online, answer peers’ academic questions, and share tips or methods for improving study efficiency. Outside of studying, many students post travel guides, shopping plans, game reviews, or promote charity projects on social media.

Focusing on online altruism at the academic level can help promote the benefits of the Internet to the public, enhance students’ mental health, reverse harmful stereotypes about the Internet, and regulate the online environment. Thus, online altruism holds significant research value.

Exploring the psychological and behavioral dimensions of university students in the era of online public opinion aids in understanding and predicting how they adapt and behave. By identifying key influencing factors such as gender, family background, economic status, and social support systems, educational institutions and families can develop effective intervention strategies. Moreover, such research provides valuable insights for policymakers and mental health professionals to design targeted mental health promotion and intervention programs, thereby enhancing the overall well-being and social adaptability of university students.

## 2 Subjects and methods

### 2.1 Data collection

The study utilized a questionnaire method, selecting a convenience sample of college students from four universities in Xuzhou, Jiangsu Province, namely Xuzhou Medical University, Jiangsu Normal University, China University of Mining and Technology, and Xuzhou University of Technology. The selection criteria included currently enrolled students aged 18–26, with diverse academic majors and year levels, to capture a broad spectrum of perspectives.

After obtaining informed consent from the participants, the online questionnaire was administered via Questionnaire Star. Prior to completing the questionnaire, students were informed that the results would be used solely for academic research, kept strictly confidential, and were provided with instructions on how to complete the questionnaire. A total of 2,398 responses were collected. After excluding invalid questionnaires with extensive blank responses, highly inconsistent answers, or excessively short completion times, 2,294 valid questionnaires were retained, resulting in an effective response rate of 95.7%. Among the valid respondents, 916 were male (39.9%) and 1,378 were female (60.1%); 1,118 were freshmen (48.7%), 579 were sophomores (25.2%), and 597 were juniors or above (26.1%); 990 were from urban areas (43.2%) and 1,304 were from rural areas (56.8%).

### 2.2 Tools for data collections

A self-administered questionnaire was employed to collect data, structured into four sections:

**Part I:** This section collected the sociodemographic information of the study participants, including gender, grade, native place, whether they are an only child, and family economic status.

**Part II:** This section focused on the influence of online public opinion on the emotions, perceptions, and behaviors of college students. It covered topics such as the duration of social media use (On average, how much time do you spend on social media each day?), whether participants believe online public opinion serves as an emotional outlet (Do you think online public opinion serves as an outlet for college students to express their emotions?), the extent to which it affects their emotional and mental health (To what extent do you think online public opinion influences college students in the following aspects? -Affect emotional and psychological health), its impact on their academic efficiency (To what extent do you think online public opinion influences college students in the following aspects? -Affect learning efficiency), and whether they have experienced strong emotional reactions to online public opinion (Have you ever had a strong emotional reaction to online public opinion?).

**Part III:** This section incorporates three scales.

#### 2.2.1 Belief in a just world scale

The just-world belief scale developed by Dalbert (1999), was utilized in this study. This scale consists of two subscales: the General Belief in a Just World Scale (GBJW) and the Personal Belief in a Just World Scale (SBJW). The GBJW subscale assesses individuals’ perceptions of the fairness of events affecting others, while the SBJW subscale evaluates individuals’ perceptions of the fairness of events affecting themselves.

The scale comprises a total of 13 items. The SBJW includes a “belief in self-justice” subscale with seven items, such as, “I believe most things that happen in my life are fair.” The GBJW includes six items, for example, “Generally, others are treated fairly.” Responses were recorded on a 6-point Likert scale, where 1 denotes complete disagreement and 6 denotes complete agreement. The average score for each item was calculated, with higher scores indicating a stronger belief in justice. In this study, the Cronbach’s *α* coefficients for the General Belief in a Just World subscale, the Personal Belief in a Just World subscale, and the overall scale were 0.917, 0.949, and 0.957, respectively.

#### 2.2.2 Connor-Davidson resilience scale

The Connor-Davidson Resilience Scale (CD-RISC) (Connor and Davidson, 2003) is used to measure the ability to cope with stress and adversity. This study used a revised version (Yu and Zhang, 2007) translated by Nan Xiao to measure the level of psychological resilience in university students. This scale contains 25 question entries divided into three dimensions of “Optimism,” “Self-improvement” and “Tenacity,” using a 5-point Likert scale: not true at all (0) to true almost all the time (Xianliang et al., 2021). The total score ranges from 0 to 100, with a higher score indicating more resilience. In this study, the Cronbach’s *α* for the scale was 0.969. The Cronbach’s α values for the three sub-dimensions: Hardiness, Strength, and Optimism were 0.951, 0.919, and 0.831, respectively.

#### 2.2.3 Internet altruistic behavior scale

This scale, developed by Zheng et al. (2022), consists of 26 items encompassing four dimensions: Online support (e.g., “giving attention and encouragement to netizens”), Online guidance (e.g., “guiding netizens on how to use the internet better”), Online sharing (e.g., “sharing your successes with others online”), and Online reminders (e.g., “informing netizens about certain online traps”). These items are scored on a 4-point scale, with 1 indicating “never” and 4 indicating “always.” Higher scores indicate a higher frequency of altruistic online behaviors in daily life. In this study, the Cronbach’s *α* for the entire scale was 0.981, and the Cronbach’s α for the subscales of Online Support, Online Guidance, Online Sharing, and Online Reminders were 0.948, 0.944, 0.939, and 0.908, respectively.

### 2.3 Data analysis

In this study, data analysis was conducted using SPSS 26.0 for descriptive statistics, including tests for common method bias, independent samples t-tests, analysis of variance (ANOVA), correlation analysis, and multiple linear regression analysis. Continuous variables were represented as means and standard deviations (SD), while categorical variables were presented as frequencies and percentages. The t-test and one-way ANOVA were employed to assess the relationships between the mean scores of the three scales and categorical variables across two or more groups in the ANOVA. Pearson’s correlation analysis was used to examine the relationships between the three scales in pairs. Multivariate analyses with the three scale scores as dependent variables were performed using multiple linear regression, incorporating only significant independent variables and excluding non-significant ones. All tests were conducted at the 0.05 level of statistical significance. In cases of missing data, any responses with incomplete data, whether total or partial, were excluded from the analyses.

## 3 Results

### 3.1 Common method bias test

The use of self-report to collect data in this study may lead to spurious correlations between variables, which in turn may affect the validity of the measure. Therefore, exploratory factor analysis was conducted on all questions using Harman’s one-way test. The results showed that there were five factors with eigenvalues greater than 1. These factors explained a total of 68.77% of the total variance, and the first factor explained 34.20% of the variance, which was less than the critical criterion of 40% (Jordan and Troth, 2020) and did not account for more than half of the total variance explained; therefore, this study did not suffer from the problem of serious common methodological bias.

### 3.2 Percentage distribution of the impact of online public opinion on college students’ emotions, cognition, and behavior

Table 1 shows that the majority of respondents (43.7%) spend 1 to 3 h on social media daily. Over half of the university students (67.3%) believe that online public opinion serves as an outlet for their emotions. 34.8% of respondents think that online public opinion has a high impact on their emotions and mental health, while 19.5% believe the impact is very high. Additionally, 30.4% think online public opinion significantly affects their learning efficiency, with 20.7% considering the impact to be very high. Lastly, 30.5% of respondents have experienced strong emotional reactions due to online public opinion.

**Table 1:** Survey results on the impact of online public opinion on college students’ emotions, cognition, and behavior (*N* = 2,294).

Variables	Frequency	Percentage (%)
Daily social media usage		
<1 h	176	7.7
1-3 h	1,003	43.7
3-5 h	701	30.6
≥5 h	414	18.0
Will online public opinion become an outlet for college students’ emotions?		
Yes	1,544	67.3
No	750	32.7
To what extent do you think online public opinion affects emotional and mental health?		
Very low impact	83	3.6
Low impact	178	7.8
Moderate impact	786	34.3
High impact	799	34.8
Very high impact	448	19.5
To what extent do you think online public opinion affects learning efficiency?		
Very low impact	89	3.9
Low impact	191	8.3
Moderate impact	840	36.6
High impact	698	30.4
Very high impact	476	20.7
Have you ever had a strong emotional reaction due to online public opinion?		
Yes	699	30.5
No	1,595	69.5

### 3.3 Analysis of factors influencing scores on the three scales

As indicated in Table 2, the overall average score of BJW is significantly associated with gender (*P* < 0.001), family economic status (*P* < 0.001), parents’ attitudes (*P* < 0.001), attention to online public opinion (*P* < 0.001), relationships with classmates (*P* < 0.001), and the degree of impact from negative or pessimistic content (*P* < 0.001). Similarly, the overall average score of CD-RISC shows significant correlations with being an only child (*P* = 0.029), family economic status, parents’ attitudes (*P* < 0.001), attention to online public opinion (*P* < 0.001), relationships with classmates (*P* < 0.001), and the degree of impact from negative or pessimistic content (*P* < 0.001). The overall average score of IAB is significantly correlated with gender (*P* < 0.001), grade level (*P* < 0.001), family economic status (*P*= 0.002), parents’ attitudes (*P* = 0.037), attention to online public opinion (*P* < 0.001), relationships with classmates (*P* = 0.001), and the degree of impact from negative or pessimistic content (*P* < 0.001).

**Table 2:** Relationship between the overall average scores of the three scales and certain Variables.

Variables	BJW		CD-RISC		IAB	
	Mean ± SD	t/F(*P*)	Mean ± SD	t/F(*P*)	Mean ± SD	t/F(*P*)
Gender						
Male	52.12 ± 11.79	−4.283***	63.26 ± 16.41	−0.028	56.65 ± 17.92	5.507***
Female	54.06 ± 9.75		63.28 ± 13.86		52.63 ± 16.54	
Grade						
First year	53.44 ± 10.83	0.576	63.56 ± 15.34	0.416	55.46 ± 17.01	7.273***
Second year	52.88 ± 10.40		63.08 ± 14.08		54.01 ± 17.96	
Third year or more	53.38 ± 10.56		62.93 ± 14.96		52.16 ± 16.67	
Native place						
Urban	53.45 ± 10.90	0.645	63.84 ± 15.41	1.587	53.96 ± 17.27	−0.678
Rural	53.16 ± 10.46		62.84 ± 14.54		54.45 ± 17.17	
Only child or not	53.28 ± 10.65		63.28 ± 14.93		54.24 ± 17.21	
Yes	53.11 ± 10.80	−0.672	64.07 ± 15.83	2.191**	54.19 ± 16.99	−0.106
No	53.41 ± 10.54		62.69 ± 14.20		54.27 ± 17.38	
Family economy						
High	54.79 ± 11.93	10.180***	69.37 ± 16.67	24.944***	58.11 ± 18.24	6.148**
Middle	53.47 ± 10.26		63.16 ± 14.41		54.07 ± 16.89	
Low	50.44 ± 12.21		59.10 ± 16.08		52.40 ± 18.57	
Parents’ attitude						
Demanding obedience from me	50.55 ± 12.50	22.111***	58.49 ± 16.55	28.426***	52.85 ± 17.73	2.828*
Demanding nothing from me	50.22 ± 10.44		58.84 ± 14.95		52.16 ± 17.66	
Willing to listen to me	53.59 ± 10.24		63.77 ± 13.49		54.87 ± 16.90	
Willing to consult with me before making a decision	54.76 ± 10.09		65.60 ± 14.76		54.83 ± 17.09	
Relationship with classmates						
Harmonious	54.31 ± 10.29	46.382***	64.97 ± 14.33	54.617***	54.93 ± 17.40	6.729***
Average	50.09 ± 10.36		57.57 ± 14.48		51.72 ± 15.97	
Difficult	40.95 ± 20.87		53.38 ± 31.01		54.48 ± 23.65	
Attention to online public opinion						
Not at all concerned	45.24 ± 19.32	7.822***	58.90 ± 28.15	7.168***	50.81 ± 21.59	8.676***
Not very concerned	52.34 ± 10.76		61.21 ± 14.54		52.82 ± 17.22	
Somewhat concerned	53.30 ± 9.96		62.51 ± 14.32		52.68 ± 16.90	
Quite concerned	54.05 ± 10.39		64.88 ± 14.12		56.31 ± 16.78	
Very concerned	53.37 ± 13.36		67.63 ± 20.11		60.71 ± 19.98	
Degree of impact from negative or pessimistic content on you						
No impact	51.12 ± 15.66	8.286***	68.94 ± 21.72	13.884***	48.09 ± 18.03	11.346***
Slight impact	53.97 ± 10.64		63.93 ± 13.75		51.95 ± 15.85	
Moderate impact	52.55 ± 8.91		61.47 ± 13.35		54.97 ± 16.18	
Significant impact	55.13 ± 9.99		63.91 ± 13.35		55.73 ± 18.04	
Severe impact	54.98 ± 15.36		66.44 ± 19.69		58.12 ± 21.95	

*P <0.05, **P <0.01, ***P <0.001.

### 3.4 Correlation analysis of various variables

Gender, grade level, only-child status, family economic status, parents’ attitude, and relationships with classmates were used as control variables to conduct a correlation analysis of belief in a just world, psychological resilience, and online altruistic behavior. The results are shown in Table 3.

**Table 3:** Correlation coefficients of various variables.

	BJW	GBJW	PBJW	CD-RISC	HAR	STR	OPT	IAB	IZ	ID	IF	IT
BJW	1											
GBJW	0.94***	1										
PBJW	0.94***	0.77***	1									
CD-RISC	0.45***	0.38***	0.47***	1								
HAR	0.42***	0.36***	0.42***	0.97***	1							
STR	0.46***	0.37***	0.48***	0.95***	0.88***	1						
OPT	0.41***	0.34***	0.44***	0.85***	0.76***	0.79***	1					
IAB	0.13***	0.16***	0.08***	0.24***	0.27***	0.16***	0.23***	1				
IZ	0.14***	0.16***	0.10***	0.25***	0.27***	0.19***	0.24***	0.97***	1			
ID	0.11***	0.16***	0.05**	0.22***	0.25***	0.13***	0.21***	0.96***	0.90***	1		
IF	0.09***	0.14***	0.04	0.20***	0.23***	0.12***	0.20***	0.93***	0.86***	0.89***	1	
IT	0.13***	0.16***	0.09***	0.24***	0.26***	0.17***	0.22***	0.94***	0.90***	0.88***	0.82***	1

BJW, GBJW, PBJW: Total score of Belief in a Just World, General Belief in a Just World, Personal Belief in a Just World; CD-RISC, HAR, STR, OPT: Total score of Psychological Resilience, Hardiness, Strength, Optimism; IAB, IZ, ID, IF, IT: Total score of Online Altruistic Behavior, Online Support, Online Guidance, Online Sharing, Online Reminders. ***P <0.001.

The total score of belief in a just world and its dimensions are significantly positively correlated with the total score of psychological resilience and its dimensions, as well as the total score of online altruistic behavior and its dimensions (except for the personal belief in a just world dimension and the online sharing dimension). The total score of psychological resilience and its dimensions are significantly positively correlated with the total score of online altruistic behavior and its dimensions (results including actual *p*-values are seen in Table 3 and Supplementary materials). These tests indicate that there are significant correlations between belief in a just world, psychological resilience, and online altruistic behavior.

### 3.5 Multiple linear regression analysis of predictor variables and scale scores in the study group

Table 4 indicates that the BJW of the study subjects can best be predicted by gender ( β =0.082, *P* <0.001), parents’ attitude ( β =0.063, *P* <0.001), relationship with classmates ( β = − 0.088, *P* <0.001), CD-RISC ( β =0.456, *P* <0.001), and the degree of impact from negative or pessimistic content ( β =0.108, *P* <0.001).

**Table 4:** Multiple linear regression analysis of some predictors and BJW scores in the study group.

Predictor variable	Unstandardized coefficients		Standardized coefficients	*t*	Sig.
	B	Std. error	Beta		
Gender	1.776	0	0.082	4.494	<0.001
Family economy	−0.311	0.459	−0.012	−0.677	0.498
Parents’ attitude	0.646	0.191	0.063	3.381	<0.001
Relationship with classmates	−2.103	0.449	−0.088	−4.682	<0.001
Attention to online public opinion	0.132	0.241	0.010	0.547	0.341
Degree of Impact from Negative or Pessimistic Content on You	1.191	0.203	0.108	5.858	<0.001
CD-RISC	0.325	0.014	0.456	23.616	<0.001
IAB	−0.001	0.012	−0.001	−0.055	0.956
Constant	27.137	1.963		13.821	<0.001

Adjusted R^2^ = 0.266, *P* < 0.001. B, beta co-efficient; SEB, standard error.

Table 5 indicates that the CD-RISC of the study subjects can best be predicted by household economic status ( β = − 0.069, *P* <0.001), parents’ attitude ( β =0.071, *P* <0.001), relationship with classmates ( β = − 0.086, *P* <0.001), attention to online public opinion ( β =0.058, *P* <0.001), degree of impact from negative or pessimistic content ( β = − 0.100, *P* <0.001), BJW ( β =0.429, *P* <0.001), and IAB ( β =0.190, *P* <0.001).

**Table 5:** Multiple linear regression analysis of some predictors and CD-RISC scores in the study group.

Predictor variable	Unstandardized coefficients		Standardized coefficients	*t*	Sig.
	B	Std. error	Beta		
Family economy	−2.441	0.623	−0.069	−3.919	<0.001
Parents’ attitude	1.019	0.260	0.071	3.926	<0.001
Relationship with classmates	−2.882	0.611	−0.086	−4.717	<0.001
Attention to online public opinion	1.076	0.327	0.058	3.290	<0.001
Degree of impact from negative or pessimistic content on you	−1.546	0.276	−0.100	−5.601	<0.001
BJW	0.601	0.025	0.429	23.663	<0.001
IAB	0.165	0.015	0.190	10.688	<0.001
Constant	28.774	2.582		11.144	<0.001

Adjusted R^2^ = 0.308, *P* < 0.001. B, beta co-efficient; SEB, standard error.

Table 6 indicates that the IAB of the study subjects can best be predicted by gender ( β = − 0.130, *P* <0.001), grade ( β = − 0.077, *P* <0.001), attention to online public opinion ( β =0.069, *P* <0.001), degree of impact from negative or pessimistic content ( β =0.146, *P* <0.001), and CD-RISC ( β =0.242, *P* <0.001).

**Table 6:** Multiple linear regression analysis of some predictors and IAB scores in the study group.

Predictor variable	Unstandardized coefficients		Standardized coefficients	t	Sig.
	B	Std. error	Beta		
Gender	−4.573	0.699	−0.130	−6.544	<0.001
Grade	−1.587	0.407	−0.077	−3.899	<0.001
Family economy	−1.601	0.816	−0.039	−1.962	0.050
Parents’ attitude	−0.107	0.341	−0.006	−0.313	0.754
Relationship with classmates	−1.028	0.802	−0.027	−1.283	0.200
Attention to online public opinion	1.475	0.428	0.069	3.448	<0.001
Degree of Impact from Negative or Pessimistic Content on You	2.601	0.360	0.146	7.232	<0.001
BJW	−0.001	0.037	−0.001	−0.030	0.976
CD-RISC	0.279	0.027	0.242	10.444	<0.001
Constant	39.063	3.620		10.790	<0.001

Adjusted R^2^ = 0.113, *P* < 0.001. B, beta co-efficient; SEB, standard error.

## 4 Discussion

This study aimed to explore the psychological and behavioral states of university students influenced by online public opinion, thus identifying key factors affecting these states. Findings indicate that a substantial portion of students (67.3%) perceive online public discourse primarily as an outlet for emotional expression. Additionally, online public opinion impacts the emotions and mental health of 34.8% of participants considerably, with 19.5% reporting a very high degree of impact. Furthermore, 30.4% of students believe that online public opinion affects their learning efficiency, while 20.7% regard the impact as very high. Notably, 30.5% have encountered strong emotional responses triggered by online public opinion. These findings underscore the necessity for further research and interventions targeting the psychological and behavioral aspects of university students in relation to online public opinion.

Key predictive variables, such as belief in a just world (BJW), psychological resilience (CD-RISC), and online altruistic behavior (IAB), were identified through analysis of factors including gender, parental attitudes, peer relationships, psychological resilience, and sensitivity to negative content. Gender emerges as a significant predictor of BJW, with female students exhibiting higher scores ( β = 0.082, *P* < 0.001), potentially reflecting a greater sensitivity to social justice and empathy (Oydemir and Dikmen, 2024; Sánchez-Prada et al., 2022; Etchezahar et al., 2022). Children’s opinions respected by parents predict higher BJW ( β = 0.063, *P* < 0.001), highlighting the importance of the family environment in shaping perceptions of justice. Adverse childhood environments are associated with psychological issues (Ningning et al., 2023), and strong parental psychological control is linked to lower BJW (Sun et al., 2023). Positive peer relationships are found to predict higher BJW scores ( β = −0.088, *P* < 0.001). Additionally, psychological resilience is significantly positively correlated with BJW ( β = 0.456, *P* < 0.001), suggesting that higher resilience enhances beliefs in a just world. Interestingly, sensitivity to negative content correlates with higher BJW ( β = 0.108, *P* < 0.001), likely owing to increased awareness and reflection on injustice.

Examining predictors of psychological resilience, this study finds significant factors including family economic status, parental attitudes, peer relationships, engagement with online public opinion, sensitivity to negative content, BJW, and IAB. Better economic conditions foster greater resilience ( β = −0.069, *P* < 0.001), aligning with existing literature (Tang et al., 2024). Parental respect for children’s viewpoints enhances resilience ( β = 0.071, *P* < 0.001), highlighting supportive family dynamics (Qi and Wu, 2024). Positive peer relationships ( β = −0.086, *P* < 0.001) and active engagement with online public opinion ( β = 0.058, *P* < 0.001) also contribute to higher resilience, suggesting the protective role of social support and information (Li et al., 2024; Wu et al., 2024). However, high sensitivity to negative content inversely affects resilience ( β = −0.100, *P* < 0.001), as negative emotions might compromise coping strategies. The results of a previous study also suggest that individuals generate emotional feedback according to the risk characteristics associated with different types of online opinions, and that risk perception is most likely to trigger negative emotions (Skagerlund et al., 2020). Higher BJW and IAB scores are also associated with higher CD-RISC scores ( β = 0.429, *P* < 0.001; β = 0.190, *P* < 0.001), suggesting that belief in a just world and moderate internet use enhance resilience.

When predicting online altruistic behavior, significant factors include gender, academic year, attention to online opinions, sensitivity to negative content, and levels of resilience (CD-RISC). Males exhibit greater online altruistic behavior (IAB) ( β = −0.130, *P* < 0.001), and higher-grade students score lower ( β = −0.077, *P* < 0.001), possibly due to academic pressures limiting time for altruistic activities. Higher levels of attention to online opinion correlate with increased altruism ( β = 0.069, *P* < 0.001), perhaps as students express their stance positively. Sensitivity to negative content relates to higher altruistic behavior (IAB) ( β = 0.146, *P* < 0.001), likely as a coping mechanism for emotional release and seeking social support. Research has found that social trust mediates the relationship between negative news and helping behavior, with excessive exposure to negative news reducing social trust and decreasing helping behavior (Han et al., 2019). Notably, CD-RISC scores are significantly positively correlated with IAB scores ( β = 0.242, *P* < 0.001), indicating that individuals with strong resilience can maintain a positive mindset in the face of stress and challenges and are more capable of engaging in altruistic behavior. These individuals can see the positive aspects of adversity and are willing to help others to promote their recovery and growth. Contrary to some studies that found BJW predicts altruistic actions (Wang et al., 2023; Jiang et al., 2017), our study does not reflect this, possibly due to demographic variations.

The study highlights the importance of peer relationships in predicting CD-RISC and BJW scores. As university students gradually separate from their parents and seek peer support, the influence of peers at school becomes stronger (Li et al., 2020). Positive peer relationships can reduce behavioral problems and improve mental health and life satisfaction (Cuicui et al., 2023), while adverse peer relationships can lead to negative emotions (Smith et al., 2014). The university environment is unique, with peer relationships at its core. In the context of online public opinion, the importance of peer relationships is even more pronounced. As online platforms become crucial for university students’ communication, online public opinion continually affects their emotions and behavior. Online interactions among peers facilitate timely sharing and discussion of information, helping each other cope with the impact of online public opinion’s impact. Positive peer relationships extend beyond offline communication to online interactions, enhancing resilience through network support and empathy. Mutual trust and understanding within peer relationships help students maintain rationality when facing complex online information, reducing the likelihood of being misled and fostering healthy beliefs. Moreover, good peer relationships enhance trust and security, making students more likely to believe in a just world (Cuicui et al., 2023). This positive interpersonal interaction experience strengthens their beliefs in fairness and justice.

Expanding the discussion on how online public opinion impacts psychological resilience, educators and policymakers can play a critical role in enhancing students’ resilience. Specific interventions could include organizing workshops and training sessions focused on developing critical thinking and emotional regulation skills. Such initiatives can empower students to identify and manage negative opinions effectively, boosting their resilience. Further, schools can create support groups where students discuss and address the effects of online public opinion collaboratively. Policymakers can implement more robust internet usage policies that protect student privacy while mitigating the spread of negative content online. Additionally, schools and universities can establish mental health resource centers that provide timely psychological support. Courses on resilience-building and online etiquette can also be integrated into the curriculum to promote healthy online habits and communication skills. Lastly, family engagement activities can be conducted to enhance the home environment’s supportive role in student mental health.

The results indicate that psychological resilience can positively predict online altruistic behavior, and online altruistic behavior can also positively predict psychological resilience. Previous research has found that for university students, high altruistic behavior can reduce the impact of negative emotions and enhance well-being (Lu et al., 2021). When individuals receive more social support from online environments, they experience more positive emotions, which facilitates altruistic behavior (Feng and Zhang, 2022). Therefore, online altruistic behavior not only significantly affects university students’ positive psychological qualities but also helps establish a virtuous cycle.

## 5 Conclusion

This study highlights how online public opinion significantly affects the psychological adaptation and behavioral performance of college students. The findings demonstrate that factors such as gender, family economic status, parents’ attitudes, relationships with classmates, and responses to negative online content significantly influence scores on the BJW, the CD-RISC, and the IAB. Significant predictors for BJW include gender, parents’ attitudes, relationships with classmates, the impact of negative content, and psychological resilience. For CD-RISC, significant predictors include family economic status, parents’ attitudes, relationships with classmates, attention to online public opinion, the impact of negative content, BJW, and IAB. Significant predictors for IAB include gender, grade level, attention to online public opinion, the impact of negative content, and psychological resilience. Overall, the study underscores the need for targeted interventions by families and schools to foster good mental health and positive behavior among college students in the digital age. Future research could benefit from longitudinal data to further explore these dynamics.

## 6 Limitations

This study presents several limitations that warrant consideration. First, the reliance on a cross-sectional design restricts our ability to establish causality between online public opinion and the psychological and behavioral responses observed in college students. Future research employing longitudinal methods could better elucidate these causal relationships over time.

Second, the study utilized a convenience sample drawn from universities in Xuzhou City, which may not fully capture the diversity of the broader college student population. This limitation affects the generalizability of our findings to other regions and cultural settings.

Third, data collection was based primarily on self-reported measures, which are inherently subject to social desirability and recall biases. Despite implementing a common method bias test, the accuracy of some responses may still be compromised.

Additionally, the small sample size of this study reduces the generalizability of our findings to a larger population.

Finally, while analysis accounted for variables such as family economic status and parental attitudes, it is important to acknowledge that other unmeasured factors—such as personal experiences with online harassment or available support systems—could significantly influence students’ psychological resilience and online behaviors. Future studies should aim to incorporate a more comprehensive array of influencing factors and consider the use of mixed-method approaches to provide deeper insights into these dynamics.

## Data Availability

The raw data supporting the conclusions of this article will be made available by the authors, without undue reservation.
