# Supplementary material for: College students’ psychology and behavior in the context of online public opinion: a cross-sectional study in Jiangsu Province, China
Source: Front Psychol. 2024 Nov 26;15:1475581. doi: 10.3389/fpsyg.2024.1475581 (PMC11628265; doi:10.3389/fpsyg.2024.1475581)
Supplement: Supplementary file 1 [file Data_Sheet_1.ZIP › Supporting information files/Supporting information questionaire.docx]

**Survey on the Basic Situation of the Influence of Online Public Opinion on College Students in the New Media Era**

Hello! We sincerely invite you to take a few minutes to fill out this survey. Your participation is anonymous, and the information you provide will be used solely for academic research. We ensure complete confidentiality of your responses. There are no right or wrong answers; please answer according to your actual situation. We greatly appreciate your cooperation.

1. What is your gender? [Single choice] *

- ○1. Male

- ○2. Female

2. The university you are currently attending: [Fill in the blank] *

_________________________________

3. Which grade are you in? [Single choice] *

- ○ First-year

- ○ Second-year

- ○ Third-year

- ○ Fourth-year

- ○ Fifth-year

- ○ Graduate student

4. Your registered residence is in [Single choice] *

- ○ Town/City

- ○ Rural area

5. Are you the only child? [Single choice] *

- ○ Yes

- ○ No

6. How would you describe your family's financial situation? [Single choice] *

- ○ Good

- ○ Average

- ○ Poor

7. You feel your parents: [Single choice] *

- ○ Require me to follow their advice

- ○ Do not have specific demands

- ○ Are willing to listen to my opinions

- ○ Are willing to make decisions after discussing with me

8. How do you find your relationship with your classmates? [Single choice] *

- ○ Harmonious

- ○ Average

- ○ Difficult

9. On average, how much time do you spend on social media each day? [Single choice] *

- ○＜1 hour

- ○ 1-3 hours

- ○ 3-5 hours

- ○ 5 hours or more

10. What is your attention towards online public opinion? [Single choice] *

- ○ Not at all concerned

- ○ Not very concerned

- ○ Somewhat concerned

- ○ Quite concerned

- ○ Very concerned

11. Do you think online public opinion serves as an outlet for college students to express their emotions? [Single choice] *

- ○ Yes

- ○ No

12. To what extent do you think online public opinion influences college students in the following aspects? The higher the score, the greater the influence. [Matrix scale] *

- Affect emotional and psychological health: ○ 1 ○ 2 ○ 3 ○ 4 ○ 5

- Affect learning efficiency: ○ 1 ○ 2 ○ 3 ○ 4 ○ 5

13. Have you ever had a strong emotional reaction to online public opinion? [Single choice] *

- ○ Yes

- ○ No

14. To what extent do negative or pessimistic online public opinions affect your emotional fluctuations? The higher the score, the greater the influence. [Single choice] *

- ○ No impact

- ○ Slight impact

- ○ Moderate impact

- ○ Significant impact

- ○ Severe impact

**15. **Belief in a Just World Scale****

Please read the following statements and make the appropriate choice. If a statement does not relate to your experience, please answer with your first impression. There are no right or wrong answers. [Matrix single choice] *

1. I think the world is basically a just place.

○ Completely disagree

○ Generally disagree

○ Somewhat disagree

○ Somewhat agree

○ Generally agree

○ Completely agree

2. To a large extent, I believe people get what they deserve.

○ Completely disagree

○ Generally disagree

○ Somewhat disagree

○ Somewhat agree

○ Generally agree

○ Completely agree

3. I am convinced that justice always triumphs over injustice.

○ Completely disagree

○ Generally disagree

○ Somewhat disagree

○ Somewhat agree

○ Generally agree

○ Completely agree

4. In the long run, I believe people who suffer from injustice will be compensated.

○ Completely disagree

○ Generally disagree

○ Somewhat disagree

○ Somewhat agree

○ Generally agree

○ Completely agree

5. I firmly believe that injustice, in various aspects of life (including career, family, politics, etc.), is accidental, not inevitable.

○ Completely disagree

○ Generally disagree

○ Somewhat disagree

○ Somewhat agree

○ Generally agree

○ Completely agree

6. I think people strive for justice when making major decisions.

○ Completely disagree

○ Generally disagree

○ Somewhat disagree

○ Somewhat agree

○ Generally agree

○ Completely agree

7. To a large extent, I believe what happens to me is what I deserve.

○ Completely disagree

○ Generally disagree

○ Somewhat disagree

○ Somewhat agree

○ Generally agree

○ Completely agree

8. I usually receive fair treatment.

○ Completely disagree

○ Generally disagree

○ Somewhat disagree

○ Somewhat agree

○ Generally agree

○ Completely agree

9. I believe I usually get what I deserve.

○ Completely disagree

○ Generally disagree

○ Somewhat disagree

○ Somewhat agree

○ Generally agree

○ Completely agree

10. Overall, what happens in my life is fair.

○ Completely disagree

○ Generally disagree

○ Somewhat disagree

○ Somewhat agree

○ Generally agree

○ Completely agree

11. Injustice happening in my life is accidental rather than inevitable.

○ Completely disagree

○ Generally disagree

○ Somewhat disagree

○ Somewhat agree

○ Generally agree

○ Completely agree

12. I believe most things that happen in my life are fair.

○ Completely disagree

○ Generally disagree

○ Somewhat disagree

○ Somewhat agree

○ Generally agree

○ Completely agree

13. I think major decisions involving myself are generally just.

○ Completely disagree

○ Generally disagree

○ Somewhat disagree

○ Somewhat agree

○ Generally agree

○ Completely agree

**16. **Psychological Resilience Questionnaire** [Matrix single choice] ***

1. I can adapt to change.

○ Never

○ Rarely

○ Sometimes

○ Often

○ Always

2. I have close, secure relationships.

○ Never

○ Rarely

○ Sometimes

○ Often

○ Always

3. Sometimes fate or God helps.

○ Never

○ Rarely

○ Sometimes

○ Often

○ Always

4. I can handle whatever comes my way.

○ Never

○ Rarely

○ Sometimes

○ Often

○ Always

5. Past successes give me confidence to face new challenges.

○ Never

○ Rarely

○ Sometimes

○ Often

○ Always

6. I can see the humor in difficult situations.

○ Never

○ Rarely

○ Sometimes

○ Often

○ Always

7. Coping with stress makes me feel stronger.

○ Never

○ Rarely

○ Sometimes

○ Often

○ Always

8. After a hardship or illness, I tend to bounce back quickly.

○ Never

○ Rarely

○ Sometimes

○ Often

○ Always

9. I believe things happen for a reason.

○ Never

○ Rarely

○ Sometimes

○ Often

○ Always

10. I do my best no matter what.

○ Never

○ Rarely

○ Sometimes

○ Often

○ Always

11. I achieve my goals.

○ Never

○ Rarely

○ Sometimes

○ Often

○ Always

12. When things seem hopeless, I don’t give up easily.

○ Never

○ Rarely

○ Sometimes

○ Often

○ Always

13. I know where to turn for help.

○ Never

○ Rarely

○ Sometimes

○ Often

○ Always

14. Under stress, I can focus and think clearly.

○ Never

○ Rarely

○ Sometimes

○ Often

○ Always

15. I like to take the lead in problem-solving.

○ Never

○ Rarely

○ Sometimes

○ Often

○ Always

16. I am not discouraged by failures.

○ Never

○ Rarely

○ Sometimes

○ Often

○ Always

17. I consider myself a strong person.

○ Never

○ Rarely

○ Sometimes

○ Often

○ Always

18. I can make unusual or difficult decisions.

○ Never

○ Rarely

○ Sometimes

○ Often

○ Always

19. I can manage unpleasant emotions.

○ Never

○ Rarely

○ Sometimes

○ Often

○ Always

20. I sometimes need to act on intuition.

○ Never

○ Rarely

○ Sometimes

○ Often

○ Always

21. I have a strong sense of purpose.

○ Never

○ Rarely

○ Sometimes

○ Often

○ Always

22. I feel in control of my life.

○ Never

○ Rarely

○ Sometimes

○ Often

○ Always

23. I enjoy challenges.

○ Never

○ Rarely

○ Sometimes

○ Often

○ Always

24. I work hard to achieve my goals.

○ Never

○ Rarely

○ Sometimes

○ Often

○ Always

25. I take pride in my achievements.

○ Never

○ Rarely

○ Sometimes

○ Often

○ Always

**17. The following statements are about some behaviors online. Please read each sentence carefully and select the option that best describes your actual situation. [Matrix single choice] ***

1. Sharing my successful study experience online

○ Never

○ Occasionally

○ Usually

○ Always

2. Answering and guiding questions online ○ Never

○ Occasionally

○ Usually

○ Always

3. Discussing issues and expressing opinions on forums ○ Never

○ Occasionally

○ Usually

○ Always

4. Sharing book reviews online

○ Never

○ Occasionally

○ Usually

○ Always

5. Sharing life experiences and insights online

○ Never

○ Occasionally

○ Usually

○ Always

6. Guiding others on how to use the internet better

○ Never

○ Occasionally

○ Usually

○ Always

7. Commenting on posts criticizing or condemning bad behavior in society

○ Never

○ Occasionally

○ Usually

○ Always

8. Posting timely, useful daily information online

○ Never

○ Occasionally

○ Usually

○ Always

9. Creating groups or forums for network communication

○ Never

○ Occasionally

○ Usually

○ Always

10. Using professional knowledge to help others online

○ Never

○ Occasionally

○ Usually

○ Always

11. Guiding others in virus prevention and removal

○ Never

○ Occasionally

○ Usually

○ Always

12. Leaving positive feedback on others' posts

○ Never

○ Occasionally

○ Usually

○ Always

13. Recommending good articles, music, and videos online

○ Never

○ Occasionally

○ Usually

○ Always

14. Exposing illegal activities online to warn others

○ Never

○ Occasionally

○ Usually

○ Always

15. Listening to others' problems and providing guidance online

○ Never

○ Occasionally

○ Usually

○ Always

16. Writing motivational articles online to inspire others

○ Never

○ Occasionally

○ Usually

○ Always

17. Informing others about internet traps

○ Never

○ Occasionally

○ Usually

○ Always

18. Reporting inappropriate content online

○ Never

○ Occasionally

○ Usually

○ Always

19. Uploading useful software programs

○ Never

○ Occasionally

○ Usually

○ Always

20. Sending data needed by others online

○ Never

○ Occasionally

○ Usually

○ Always

21. Offering care and encouragement to others online

○ Never

○ Occasionally

○ Usually

○ Always

22. Warning others of scams and inappropriate content online

○ Never

○ Occasionally

○ Usually

○ Always

23. Sending good wishes to others online

○ Never

○ Occasionally

○ Usually

○ Always

24. Posting about being scammed to alert others

○ Never

○ Occasionally

○ Usually

○ Always

25. Helping others resolve problems online, including life, study, and emotional issues

○ Never

○ Occasionally

○ Usually

○ Always

26. Providing technical or methodological guidance online to help novices learn internet skills

○ Never

○ Occasionally

○ Usually

○ Always
